# Insulin-like growth factor 1 receptor affects the survival of primary prostate cancer patients depending on *TMPRSS2-ERG* status

**DOI:** 10.1186/s12885-017-3356-8

**Published:** 2017-05-25

**Authors:** Caterina Mancarella, Irene Casanova-Salas, Ana Calatrava, Maria García-Flores, Cecilia Garofalo, Andrea Grilli, José Rubio-Briones, Katia Scotlandi, José Antonio López-Guerrero

**Affiliations:** 1 CRS Development of Biomolecular Therapies, Experimental Oncology Laboratory, Rizzoli Orthopedic Institute, via di Barbiano, 1/10, 40136 Bologna, Italy; 2 Laboratory of Molecular Biology, Fundación Instituto Valenciano de Oncología, C/ Prof. Beltrán Báguena, 8, 46009 Valencia, Spain; 3 Department of Pathology, Fundación Instituto Valenciano de Oncología, C/ Prof. Beltrán Báguena, 8, 46009 Valencia, Spain; 4 Department of Urology, Fundación Instituto Valenciano de Oncología, C/ Prof. Beltrán Báguena, 8, 46009 Valencia, Spain

**Keywords:** Insulin-like growth factor 1 receptor, Prostate cancer, *TMPRSS2-ERG*, Prognosis, Molecular biotypes

## Abstract

**Background:**

Prostate cancer (PCa) is characterized by clinical and biological heterogeneity and has differential outcomes and mortality rates. Therefore, it is necessary to identify molecular alterations to define new therapeutic strategies based on the risk of progression. In this study, the prognostic relevance of the insulin-like growth factor (IGF) system was examined in molecular subtypes defined by *TMPRSS2-ERG* (T2E) gene fusion within a series of patients with primary localized PCa.

**Methods:**

A cohort of 270 formalin-fixed and paraffin-embedded (FFPE) primary PCa samples from patients with more than 5 years’ follow-up was collected. *IGF-1R*, *IGF-1*, *IGFBP-3* and *INSR* expression was analyzed using quantitative RT-PCR. The T2E status and immunohistochemical ERG findings were considered in the analyses. The association with both biochemical and clinical progression-free survival (BPFS and PFS, respectively) was evaluated for the different molecular subtypes using the Kaplan-Meier proportional risk log-rank test and the Cox proportional hazards model.

**Results:**

An association between *IGF-1R* overexpression and better BPFS was found in T2E-negative patients (35.3% BPFS, *p*-value = 0.016). Multivariate analysis demonstrated that *IGF-1R* expression constitutes an independent variable in T2E-negative patients [HR: 0.41. CI 95% (0.2–0.82), *p* = 0.013]. These data were confirmed using immunohistochemistry of ERG as subrogate of T2E. High *IGF-1* expression correlated with prolonged BPFS and PFS independent of the T2E status.

**Conclusions:**

*IGF-1R*, a reported target of T2E, constitutes an independent factor for good prognosis in T2E-negative PCa. Quantitative evaluation of *IGF-1*/*IGF-1R* expression combined with molecular assessment of T2E status or ERG protein expression represents a useful marker for tumor progression in localized PCa.

**Electronic supplementary material:**

The online version of this article (doi:10.1186/s12885-017-3356-8) contains supplementary material, which is available to authorized users.

## Background

Prostate cancer (PCa) is the most common cancer in men and the sixth cause of cancer death worldwide [1]. PCa is difficult to manage as it shows a spectrum of risk over time spanning from indolent tumors, which can be controlled with surgery or active surveillance, to tumors with aggressive and metastatic behavior that require more radical treatment strategies [2]. Consequently, there is an urgent clinical need for tools that can discriminate between the different conditions and stratify patients at diagnosis according to tumor progression risk. Established clinical and pathological prognostic factors, including serum PSA levels, Gleason score, lymph node involvement and the pathological stages of affected surgical margins, have proven useful but are insufficient for optimal risk stratification. From the genetic point of view, PCa can be considered a collection of cancers characterized by sets of molecular alterations that may underlie the clinically variable behavior of the disease and support the need to identify subgroups of patients with different prognoses [3]. Recently, the prognostic value of many molecular and genetic factors has been investigated, including the loss of *PTEN* or *Akt* mutations [4–6]. The prognostic significance of the *TMPRSS2-ERG* (T2E) fusion gene, a specific chromosomal rearrangement found in 50–70% of PCa that involves the androgen-responsive promoter of *TMPRSS2* and the ETS transcription factor family gene *ERG,* has been evaluated, but the results are not conclusive [7–10]. The recent application of deep-sequencing techniques has led to a more comprehensive genomic portrait of localized and potentially curable PCa [11–13], further pointing out the multifocal genetic nature of PCa and the presence of intra- and inter-tumor molecular heterogeneity that may affect tumor progression and response to therapy [14].

In the past years, several studies have recognized the prognostic role of some components of the insulin-like growth factor (IGF) system. The IGF system is composed of three receptors [insulin receptor (INSR), IGF-1 receptor (IGF-1R) and mannose 6-phosphate receptor (M6P/IGF-2R)], three ligands (insulin, IGF-1, IGF-2), and six known types of circulating IGF-binding proteins (IGFBP1–6) that modulate the bioavailability and bioactivity of the IGFs [15]. The IGF system has been reported to regulate normal and malignant growth, proliferation and differentiation, tissue homeostasis and cellular metabolism. The relevance of the IGF system and particularly IGF-1R in cancer has been widely documented [16]. The first evidence regarding the IGF system’s role in PCa came from epidemiological studies and showed that higher serum IGF-1 concentrations and decreased circulating IGFBP-3 are correlated with an increased risk of developing PCa [17]. In the prostate, IGF-1R plays a critical role in normal gland growth and development [18]. However, existing data regarding IGF system expression and its functional role in PCa are still controversial [19–21]. Clinical studies evaluating the prognostic potential of IGF-1R are limited and report either positive or negative associations between IGF-1R expression levels and patient outcome [22, 23]. In this paper, we analyzed the expression of different components of the IGF system and their association with clinico-pathological parameters and the prognosis of biochemical progression-free survival (BPFS) and clinical progression-free survival (PFS) in a retrospective series of 270 patients with primary localized PCa treated with radical prostatectomy. In a previous study, we demonstrated that the IGF system is influenced by T2E as ERG directly binds the *IGF-1R* gene promoter, thus affecting its expression in PCa [24]. This paper shows for the first time that patients with PCa who do not harbor the T2E rearrangement and who express low levels of *IGF-1R* represent a subgroup of primary PCa tumors with poor outcome.

## Methods

### Clinical prostate specimens

Formalin-fixed and paraffin-embedded (FFPE) blocks corresponding to radical prostatectomy specimens from 270 PCa patients were retrieved from the archives of the Biobank of the *Fundación Instituto Valenciano de Oncología* according to the following criteria: specimens obtained from radical retropubic prostatectomies from 1996 to 2002 and no history of previous treatment for PCa (including androgen deprivation therapy or chemotherapy prior to surgery), as previously reported [25]. The clinico-pathological features of the PCa samples analyzed in the study, including the T2E status, are summarized in Table 1. T2E gene fusion status was determined using reverse transcription polymerase chain reaction (RT-PCR) and fluorescent in situ hybridization (FISH), as previously described [10], and quantitative RT-PCR (qRT-PCR) as previously reported [25]. Briefly, cases that presented the rearrangement based on any of the three procedures (FISH, RT-PCR, qRT-PCR) were considered positive. All the patients gave written informed consent for tissue donation for research purposes before tissue samples were collected, and the study was approved by FIVO’s Institutional Ethical Committee (ref. number 2010–19). The combined Gleason score was uniformly determined by the same uro-pathologist (Ana Calatrava), who also certified the high-density cancer areas in hematoxylin and eosin-stained slides to ensure a purity of at least 75% of cancer cells. For comparative and calibration purposes, we also analyzed 8 samples of normal prostate tissue obtained from patients undergoing radical cystectomies without pathological evidence of prostate disease. Follow-up of the retrospective series ranged from 1 to 189 months (median 69 months). Biochemical progression (BPFS) was defined as serum PSA greater than 0.4 ng/ml during follow-up, and clinical progression (PFS) was defined as local (prostatic fossa), regional (lymph nodes) or distant (metastasis) progression.

**Table 1:** Clinico-pathological features of the patients included in the study

Parameter	qRT-PCR (*n* = 270)		IHC (*n* = 239)	
	No. Pts	%	No. Pts	%
Age				
≤ 55	15	5.6	12	5
56-65	81	30	72	30.1
66-75	138	51.1	122	51
> 75	36	13.3	33	13.8
Gleason-sp				
2-6	109	40.4	87	36.4
7	129	47.8	123	51.5
Greater than 7	32	11.9	29	12.1
PSA (ng/ml)				
10 or less	155	57.6	133	55.9
10-20	74	27.5	69	29
Greater than 20	40	14.9	36	15.1
cT				
cT2b or less	248	92.2	219	92
cT3a or greater	21	7.8	19	8
pT				
pT2 or less	135	50	115	48.1
pT3 or greater	135	50	124	51.9
pN^a^				
pN0	236	95.2	209	95.4
pN1 or greater	12	4.8	10	4.6
Margins				
Negative	137	50.7	116	48.5
Positive	133	49.3	123	51.5
TMPRSS2/ERG^b,c^				
Negative	92	34.1	105	48.8
Positive	178	65.9	110	51.2

SP, specimen; cT, clinical stage; PSA, prostatic specific antigen; pN, lymphnode pathological stage

^a^Lymphadenectomy was limited to the obturator fossa in most of the cases at the inclusion period.

^b^Values in qRT-PCR columns refer to TMPRSS2-ERG status determined using reverse transcription polymerase chain reaction (RT-PCR), fluorescent in situ hybridization (FISH), and quantitative RTPCR (qRT-PCR); values in IHC columns refer to immunohistochemical ERG evaluation

^c^IHC ERG expression was not detectable in 24/239 cases

### Gene expression analysis

RNA isolation was performed from three 20-μm-thick sections of FFPE tissues using RecoverAll™ Total Nucleic Acid Isolation Kit (Ambion) following the manufacturer’s specifications. RNA with a 260/280 nm absorbance ratio of 1.5–2 was reverse transcribed with the High Capacity cDNA Reverse Transcription Kit (Applied Biosystems) according to the manufacturer’s indications. Predesigned TaqMan probes (Applied Biosystems) for the target genes were used to determine their expression level using quantitative RT-PCR (qRT-PCR) and an ABI 7500-Fast Thermocycler Sequence Detection System (Applied Biosystems). The probes included *IGF-1R* (Hs00181385_m1), *INSR* (Hs00961560_m1), *IGFBP-3* (Hs00426287_m1), *IGF-1* (Hs00153126_m1), *IGF-2* (Hs04188276_m1), and *T2E* (Hs03063375_ft). For endogenous control, *β-2-microglobulin* (Hs99999907_m1) was used (Applied Biosystems). cDNA from normal human prostate samples was used as a calibrator for comparative analyses of the PCa cases. Two replicates per gene were considered. Relative quantification analysis was determined using the mean value of the control samples and the 2^-ΔΔCt^ method [26].

### Immunohistochemistry

The FFPE PCa specimens were incorporated into 11 tissue microarrays (TMA). Two or three representative areas (1 mm in diameter) of each tumor were selected for TMA production by first examining the hematoxylin and eosin-stained prostatectomy tumor slides and then sampling tissue from the corresponding paraffin blocks. A tissue microarray instrument (Beecher Instruments) was used for TMA assembly. All the cases included in the different TMAs underwent immunohistochemistry (IHC) analysis under the same conditions after the optimization of a protocol developed at the Instituto Valenciano de Oncología that ensured absence of background noise derived from the staining technique. Within each TMA section, a series of positive (tonsil) and negative controls (secondary antibody alone) were included. Three-μm-thick sections from the TMA blocks were stained using anti-human ERG clone EP111 monoclonal-Ab (Dako), and the percentage of ERG-positive cells was evaluated. The median percentage of stained cells was calculated. Cases were scored as ERG-negative when the percentage of stained cells was less than the median value and ERG-positive when the percentage of stained cells was equal to or more than median value. The clinico-pathological features of the PCa samples analyzed in the study are summarized in Table 1.

### Statistical analysis

The association between gene expression levels and clinico-pathological parameters (categorical) was assessed using Fisher’s exact test or the chi-square test, as appropriate. The impact of biological factors on BPFS and PFS was determined using Kaplan-Meier curves and the log-rank test. BPFS and PFS were considered individually from the date of surgery to the date of the event. Candidate predictors of BPFS and PFS were entered into a Cox proportional hazard model using stepwise selection to identify significant outcome predictors. The 95% confidence intervals (CI) of hazard ratios (HRs) are provided [27]. Statistical analyses were performed with SPSS® software, version 20.0. *P*-values less than or equal to 0.05 were considered significant.

## Results

### Gene expression profile of the IGF system in primary prostate cancer and its association with prognosis

**Fig. 1:**
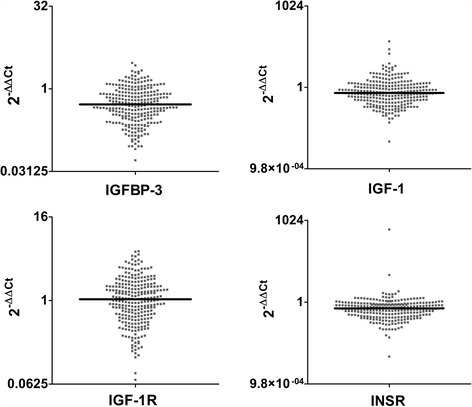
IGF system expression profile in PCa. The differential expression of *IGFBP-3*, *IGF-1*, *IGF-1R* and *INSR* between PCa and prostate normal tissues was analyzed in 270 FFPE primary PCa samples using qRT-PCR following the 2^-ΔΔCt^ method. Black lines mark the median values

**Fig. 2:**
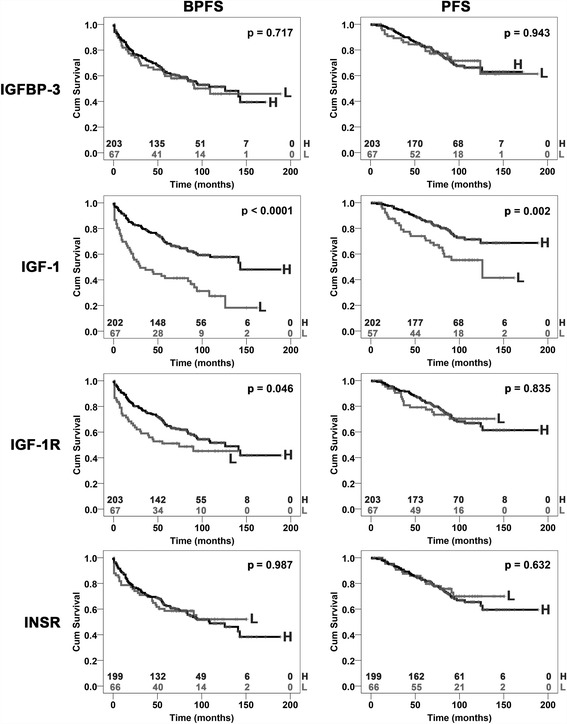
Prognostic value of *IGFBP-3*, *IGF-1*, *IGF-1R* and *INSR* for the survival of PCa patients. High-expression (H) and low-expression (L) samples were defined according to the first quartile values of RQ. Survival curves were compared using the log-rank test. Time scale refers to months from diagnosis. BPFS, biochemical progression-free survival; PFS, clinical progression-free survival. The number of patients at risk among the high- (top) and low-expression (bottom) samples are listed above each time interval

The expression of insulin-like growth factor-1 receptor (*IGF-1R*), insulin receptor (*INSR*), insulin-like growth factor-1 (*IGF-1*), insulin-like growth factor-2 (*IGF-2*) and insulin-like growth factor-binding protein-3 (*IGFBP-3*) in a retrospective series of 270 primary prostate cancer (PCa) specimens was evaluated using quantitative RT-PCR (qRT-PCR) (Fig. 1) and compared with normal prostate tissues. No *IGF-2* expression was detected in any of the analyzed cases. As previously reported [24], no differential expression compared with the normal prostate was found for *IGF-1R* (median = 1.04; range = 0.07–5.12); 48.9% of the patients showed a relative quantity (RQ) expression less than 1. *INSR* was substantially down-regulated in the tumor samples (median = 0.58; range = 0.01–471.75; RQ < 1 in 84.2% of cases), as was *IGFBP-3* (median = 0.52; range = 0.05–2.96; RQ < 1 in 80.7% of cases). A weak down-regulation of *IGF-1* was observed (median = 0.61; range = 0.01–50.12; RQ < 1 in 69.1% of cases). Patients were classified as high- or low-expressers, depending on whether the obtained RQ values were above or below the first quartile (Additional file 1) and relation with patient prognosis was evaluated using Kaplan-Meier survival curves and log-rank tests (Table 2). The median follow-up periods of the series were 69 months (from 1 to 189 months) and 82 months (from 1 to 189 months) for biochemical progression-free survival (BPFS) and progression-free survival (PFS), respectively. The analysis revealed a statistically significant association between high *IGF-1* expression and a better BPFS or PFS, while a trend in the statistical association was observed between high *IGF-1R* expression and a better BPFS (Fig. 2). According to other studies [28], *TMPRSS2-ERG* (T2E) rearrangements did not provide any information from a prognostic point of view. Multivariate analysis confirmed the statistical value of *IGF-1* as predictor of good prognosis for BPFS [HR = 0.60. CI 95% (0.39–0.90), *p* = 0.015] (Table 2). The association between *IGF-1R, INSR*, *IGF-1*, and *IGFBP-3* expression and clinico-pathological characteristics was analyzed (Additional file 2). In addition to the previously reported association between *IGF-1R* and T2E expression indicating that patients harboring the fusion gene show higher *IGF-1R* mRNA levels than T2E-negative cases (*p*-value = 0.008, Fisher’s test) [24], *IGF-1* expression was decreased in advanced PCa cases (Gleason score 7 or greater, *p*-value < 0.0001; PSA 10 ng/ml or greater, *p*-value = 0.01; clinical stage (cT) 3a or greater, *p*-value = 0.001; pathological stage (pT) 3 or greater, *p*-value = 0.005; lymphnode pathological stage (pN) 1 or greater, *p*-value < 0.0001; Fisher’s or chi-square tests**)**.

**Table 2:** BPFS and PFS log rank and Cox regression tests in primary PCa analyzed by qRT-PCR

Total cases		Biochemical progression				Clinical Progression			
Parameter	*n*	Events (% BPFS)	*p*-Univariate	HR (95% CI)	*p*-Multivariate	Events (% PFS)	*p*-Univariate	HR (95% CI)	*p*-Multivariate
Age			0.165				0.379		
≤ 55	15	5 (73.3)				3 (79.4)			
56-65	81	43 (25.7)				29 (50.1)			
66-75	138	58 (45.4)				34 (69.8)			
> 75	36	18 (48.6)				8 (59.2)			
Gleason-sp			< 0.0001		0.001		< 0.0001		0.015
2-6	109	35 (56.4)		1		17 (77.3)		1	
7	129	63 (29.8)		2.94 (1.64-5.26)	< 0.0001	43 (57.4)		3.03 (1.4-6.53)	0.005
Greater than 7	32	25 (11.7)		1.93 (1.18-3.16)	0.008	14 (0)		1.57 (0.83-2.96)	0.163
PSA (ng/ml)			< 0.0001		0.011		0.09		
10 or less	155	57 (47.8)		1		35 (68.6)			
10-20	74	37 (39.2)		2.12 (1.29-3.48)	0.003	24 (53.6)			
Greater than 20	40	29 (24.4)		1.72 (1.03-2.88)	0.036	15 (57.1)			
cT			< 0.0001		0.013		0.029	1	0.002
cT2b or less	248	107 (43)		1		66 (63.1)		2.46 (1.38-4.4)	
cT3a or greater	21	16 (14.5)		1.72 (1.03-3.67)		8 (58.6)			
pT			< 0.0001		NS		0.001		NS
pT2 or less	135	43 (57.4)				25 (77.9)			
pT3 or greater	135	80 (23)				49 (48.4)			
pN			< 0.0001		0.043		0.2		
pN0	236	104 (43)		1		64 (63.6)			
pN1 or greater	12	11 (8.3)		1.98 (1.02-3.84)		5 (50.9)			
Margins			< 0.0001		0.001		< 0.0001		0.039
Negative	137	40 (56)		1		24 (77)		1	
Positive	133	83 (21.4)		2.14 (1.39-3.32)		50 (41.6)		1.74 (1.02-2.95)	
TMPRSS2-ERG			0.105				0.957		
Negative	92	49 (32.5)				26 (63.9)			
Positive	178	74 (45.2)				48 (61.6)			
*IGF-1R*			0.046		NS		0.835		
Low	67	34 (45.3)				17 (70.3)			
High	203	89 (41.9)				57 (61.4)			
*INSR*			0.987				0.632		
Low	66	29 (52.1)				17 (69.9)			
High	199	92 (38.3)				57 (59.5)			
*IGF-1*			< 0.0001		0.015		0.002		NS
Low	67	44 (18.2)		1		26 (41.5)			
High	202	78 (48)		0.60 (0.39-0.90)		47 (68.6)			
*IGFBP-3*			0.717				0.943		
Low	67	31 (45.9)				17 (61.3)			
High	203	92 (39.5)				57 (62.9)

### Clinical relevance of IGF system components in T2E molecularly defined prostate cancer

**Fig. 3:**
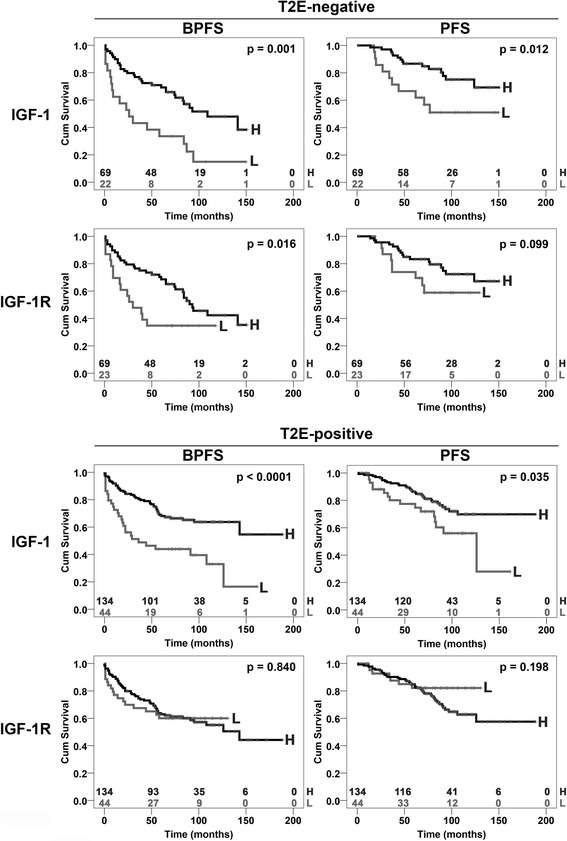
Prognostic value of *IGF-1* and *IGF-1R* in subtypes of PCa patients defined by T2E. First quartile values of RQ were separately calculated for the T2E–negative (top) and T2E-positive (bottom) groups, and the samples were classified as high-expressers (H) and low-expressers (L). Time scale refers to months from diagnosis. Black lines indicate high-expression patients. BPFS, biochemical progression-free survival; PFS, clinical progression-free survival. The number of patients at risk in the high- (top) and low-expression (bottom) samples is listed above each time interval

Since *IGF-1R* was associated with the presence of T2E [24], our series was divided according to the T2E fusion gene status. Thus, two cohorts of patients were identified: a T2E–negative (92 cases) and a T2E-positive (178 cases) cohort (Additional file 1). For each cohort, patients were defined as high- or low-expressers according to first quartile RQ values. We found that *IGF-1R* expression was decreased in advanced T2E-negative PCa cases (pT3 or greater, *p*-value = 0.05, Fisher’s exact test; Additional files 3 and 4). In the T2E-negative subgroup, the median follow-up was 65 months (from 1 to 151 months) or 82 months (from 2 to 151 months) for BPFS and PFS, respectively. In the T2E-positive subgroup, the median follow-up was 70 (from 1 to 189 months) or 81 months (from 1 to 189 months) for BPFS and PFS, respectively. Log-rank test analysis showed that *IGF-1R* was a significant predictor of prognosis in T2E-negative patients (*p*-value = 0.016, Additional file 5) but not in T2E-positive patients (Additional file 6); additionally, low *IGF-1R* expression conferred a worse prognosis for BPFS in T2E-negative patients (Fig. 3). Multivariate analysis showed that high *IGF-1R* represented a significant predictor of good prognosis in the T2E-negative cohort [HR: 0.41. CI 95% (0.2–0.82), *p* = 0.013] (Additional file 5). *IGF-1* expression was decreased in the T2E-negative advanced PCa cases (cT3a or greater, *p*-value < 0.0001; pN1 or greater, *p*-value = 0.037; Fisher’s exact test; Additional file 3) and in the T2E-positive cases (pT3 or greater, *p*-value = 0.037; pN1 or greater, *p*-value = 0.004; Fisher’s exact test; Additional file 4). *IGF-1* was associated with BPFS and PFS in both T2E subgroups (Fig. 3). The multivariate analysis showed that *IGF-1* constituted a prognostic factor regardless of the T2E status [T2E-positive: HR = 0.47. CI 95% (0.27–0.79), *p* = 0.005; T2E-negative: HR = 0.49. CI 95% (0.24–0.98), *p* = 0.045] (Additional files 5 and 6).

### ERG immunohistochemistry correlates with the molecular detection of T2E status

**Fig. 4:**
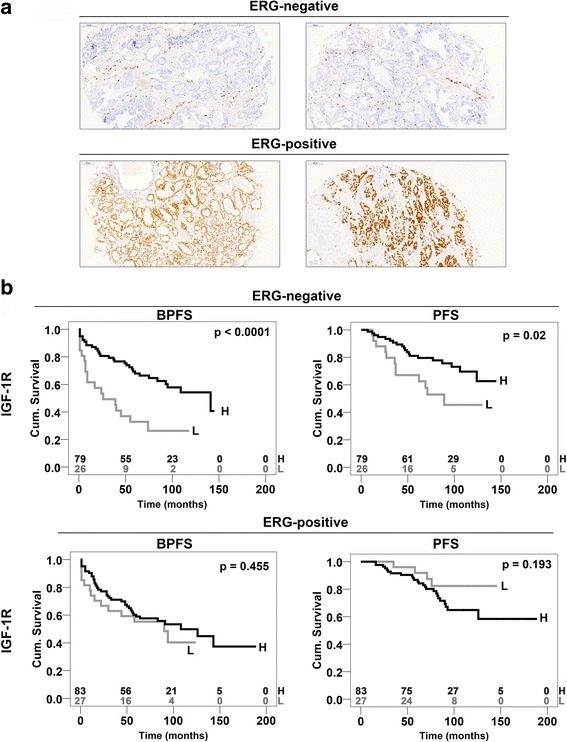
Immunohistochemical evaluation of ERG expression in 239 PCa specimens. **a**, Representative immunohistochemistry images for ERG with low expression (ERG-negative, top panels) and high expression (ERG-positive, bottom panels) in PCa tissue array samples (magnification, ×20); **b**, the first quartile value of *IGF-1R* RQ was separately calculated for the ERG–negative (top) and ERG-positive (bottom) groups, and the samples were classified as high-expression (H) and low-expression (L). Time scale refers to months from diagnosis. Black lines indicate high-expressing patients. BPFS, biochemical progression-free survival; PFS, clinical progression-free survival. The number of patients at risk in the high- (top) and low-expression (bottom) samples is listed above each time interval

Considering the existence of reliable ERG antibodies, immunohistochemistry (IHC) analysis was performed on 239 PCa samples (Additional file 7) from the same series of 270 cases to assess whether ERG IHC evaluation could be used as a surrogate marker for molecular T2E detection (Table 1). The patients were divided according to ERG protein expression levels, and two groups of patients were identified: an ERG-negative group (105 cases, Additional file 8) and an ERG-positive group (110 cases, Additional file 9; Fig. 4a). The T2E expression in each tumor was measured as reported in the Methods section and then compared to the ERG IHC status. A statistically significant correlation between ERG protein expression and T2E status (*p*-value < 0.0001; Fisher’s test) was found, thus showing that ERG IHC can serve as a surrogate marker for T2E rearrangement**.** ERG did not represent a prognosis biomarker in our series (Additional file 7).

### ERG immunohistochemistry identifies the subgroup of ERG-negative prostate cancer patients where *IGF-1R* influences prognosis

Using ERG IHC, we performed a log-rank analysis of *IGF-1R* in the ERG-negative (Additional file 8) and ERG-positive (Additional file 9) subpopulations. Cases were defined as high- or low-expressers depending on whether the obtained *IGF-1R* RQ values calculated for each cohort of patients were above or below the first quartile. The median durations of follow-up for the ERG-negative subgroup were 60 months (from 1 to 145 months) and 77 months (from 2 to 145 months) considering BPFS and PFS, respectively. The median durations of follow-up for the ERG-positive subgroup were 70 months (from 1 to 189 months) or 82 months (from 9 to 189 months) considering BPFS and PFS, respectively. The analyses confirmed that high *IGF-1R* gene expression was associated with a good prognosis in the ERG-negative patients with statistically longer BPFS and PFS (*p*-value < 0.0001 and *p*-value = 0.02, respectively) compared with those with low *IGF-1R* gene expression; however, *IGF-1R* was not associated with survival in the ERG-positive subgroup (*p*-value > 0.5; Fig. 4b). Multivariate analysis showed that *IGF-1R* represented a significant predictor of good BPFS [HR = 0.30. CI 95% (0.16–0.57), *p* = 0.001] in ERG-negative patients. The association between *IGF-1R* and clinico-pathological parameters in these subgroups of patients is shown in Additional files 10 and 11.

## Discussion

Although the relationship between the IGF axis and PCa risk and progression has been extensively studied, consensus is still needed. The discordance among studies is putatively due to different factors including i) composition of the analyzed series, ii) technical bias and iii) disregarded molecular mechanisms influencing IGF activity.

The findings reported in this study support a relationship between high *IGF-1* and *IGF-1R* mRNA expression and favorable outcomes. Overall, the results are in contrast with the common view of IGF-1R as a marker of aggressiveness; however, previous studies of sarcomas and carcinomas reported similar results. In Ewing sarcoma, lower IGF-1 circulating levels were found in patients with metastatic disease [29], while in a cohort of 57 patients, a relationship between high *IGF-1R* and *IGF-1* expression and favorable prognosis was found [30]. In breast cancer, lower expression of IGF-1R was found in tumor specimens than in matched control samples [31], and positive IGF-1R expression was associated with favorable prognosis [32]. Hence, our results are in line with evidence that the IGF-1R/IGF-1 axis is not an oncogenic driver in primary PCa. Plymate et al. demonstrated that restored expression of IGF-1R in malignant prostate cells slowed down growth both in vitro and in vivo [33], while an in vivo study by Sutherland et al. showed that conditional prostate-specific *IGF-1R* knockout caused cell proliferation, hyperplasia and the emergence of aggressive PCa when p53 activity was compromised [34]. Furthermore, Massoner et al. demonstrated that the IGF axis is up-regulated during normal epithelial differentiation in vitro [35]. In this study, we confirmed that up-regulation of *IGF-1*/*IGF-1R* signaling in local PCa is associated with a less aggressive phenotype.

Although recent advancements in next-generation sequencing technology have improved our understanding of the biology of prostate tumors [12], emphasizing the genetic basis of clinical variability of the disease, the impact of the molecular heterogeneity of PCa on the IGF axis has never been considered at clinical level. The genetic heterogeneity of PCa has become recently clear, and the molecular classification of PCa is helping to move towards a direct application of the personalized medicine concept. In this context, the presence of tumor-specific chromosomal translocations may have a crucial role. In 2005, Tomlins et al. first described the rearrangements of the ETS family of transcription factors (*TMPRSS2-ERG*) in approximately 50% of all PCa patients [36]. Since their discovery, these fusion genes have represented a powerful diagnostic biomarker. However, the prognostic significance of T2E is still controversial. Several authors have suggested an association between T2E and more aggressive tumor behavior and poor prognosis [37, 38]. In contrast, other studies have reported an association between T2E and favorable outcome, and still others did not find any association between T2E and patient survival [10, 39, 40]. In this study, we did not identify any prognostic relevance for the expression of T2E, a finding that is in line with a recent study that enrolled more than 1000 patients [41]. Nevertheless, when analyzing the expression of IGF system components according to the presence or absence of the T2E rearrangement, a difference in the value of *IGF-1R* expression as an indicator of disease progression was observed. Interestingly, these data were obtained not only dividing patients according to T2E status established using gold standard methods (PCR methods and/or FISH) but also as a result of ERG IHC evaluation. Accordingly to other studies [8, 42, 43], ERG IHC evaluation represents a reliable surrogate for T2E detection and is simpler and cheaper than molecular techniques. T2E was previously reported to influence the prognostic value of other genes. In this context, the prognostic value of *SPOP* was found to be statistically significant in the subgroup of patients not expressing the fusion gene [25]. In another study, high *NBS1* gene expression was associated with BPFS in a subgroup of T2E-negative and PTEN non-deleted PCa patients [44]. The dependence of *IGF-1R*’s prognostic value on T2E status may partly explain the controversial evidence regarding the role of IGF-1R in PCa progression. The cellular genetic background may be relevant to modulate IGF-1R signaling and functions. In fact, IGF-1R functions may be affected by complex cross-talk with other signaling pathways and by direct interactions of IGF-1R with other cell surface receptors, such as the recently discovered connection with discoidin domain receptor 1 [45]. Several papers have demonstrated that aberrant expression of ERG alters the cellular transcriptional pattern, conferring a new phenotype characterized by an increased proliferation rate and/or invasiveness and decreased differentiation levels [46–48]. The association between the IGF system and T2E rearrangement is not yet completely understood. The SWI/SNF chromatin remodeling complex, which high expression correlates with a prolonged disease-free survival in PCa patients, was demonstrated to both down-regulate transcription of *TMPRSS2* and therefore the fusion gene and to sustain *IGF-1* expression [49–51]. Recent evidences demonstrated an interaction between the IGF system and T2E by identifying *IGF-1R* as a direct target of T2E [24, 52]. The reported clinical evidence indicates that T2E, with its wide spectrum of alterations, may counteract the putative beneficial effects conferred by IGF-1R expression, likely addressing cancer cells toward a less differentiated, more aggressive phenotype.

## Conclusions

The results of this study provide new criteria for the classification of primary PCa patients based on contemporary assessment of T2E and quantification of *IGF-1R* expression. Particularly, the combination of an absence of T2E and low expression of *IGF-1R* identifies a group of patients with a poor prognosis who could benefit from a more severe treatment regimen. In addition, the data suggest an economic approach to patient stratification based on IHC ERG and *IGF-1R* evaluation. These results further support the importance of T2E for classifying the distinct biological entities associated with different risks of progression and prognosis. In conclusion, we believe these results provide a path toward more precisely establishing specific subtypes of PCa with distinct outcomes.

## Additional files

Additional file 1: *IGFBP-3*, *IGF-1*, *IGF-1R* and *INSR* RQ values of 270 PCa cases and different T2E-defined molecular subtypes. (XLS 79 kb)

Additional file 2: Association between IGF system components and clinico-pathological parameters according to Fisher’s or chi-square tests (when more than 2 categories were present) in 270 cases. (DOC 39 kb)

Additional file 3: Association between IGF system components and clinico-pathological parameters according to Fisher’s or chi-square tests (when more than 2 categories were present) in T2E-negative cases. (DOC 38 kb)

Additional file 4: Association between IGF system components and clinico-pathological parameters according to Fisher’s or chi-square tests (when more than 2 categories were present) in T2E-positive cases. (DOC 38 kb)

Additional file 5: BPFS and clinical PFS log-rank and Cox regression tests in T2E-negative PCa patients analyzed with qRT-PCR. (DOC 80 kb)

Additional file 6: BPFS and clinical PFS log-rank and Cox regression tests in T2E-positive PCa patients analyzed with qRT-PCR. (DOC 83 kb)

Additional file 7: BPFS and clinical PFS log-rank and Cox regression tests in primary PCa patients analyzed with IHC. (DOC 72 kb)

Additional file 8: BPFS and clinical PFS log-rank and Cox regression tests in ERG-negative PCa patients analyzed with IHC. (DOC 69 kb)

Additional file 9: BPFS and clinical PFS log-rank and Cox regression tests in ERG-positive PCa patients analyzed with IHC. (DOC 71 kb)

Additional file 10: Association between *IGF-1R* and clinico-pathological parameters according to Fisher’s or chi-square tests (when more than 2 categories were present) in ERG-negative cases. (DOC 32 kb)

Additional file 11: Association between *IGF-1R* and clinico-pathological parameters according to Fisher’s or chi-square tests (when more than 2 categories were present) in ERG-positive cases. (DOC 32 kb)

## Abbreviations

BPFS: Biochemical progression-free survival;
CI: Confidence interval
cT: Clinical stage
FFPE: Formalin-fixed and paraffin-embedded
FISH: Fluorescent in situ hybridization
HR: Hazard ratio
IGF: Insulin-like growth factor
IGF-1R: IGF-1 receptor
IGFBP-3: IGF-binding protein 3
IHC: Immunohistochemistry
INSR: Insulin receptor
PCa: Prostate cancer
PFS: Clinical progression-free survival
pN: Lymph node pathological stage
PSA: Prostate specific antigen
pT: Pathological stage
qRT-PCR: Quantitative RT-PCR
RQ: Relative quantity
SP: Specimen
T2E: *TMPRSS2-ERG*
TMA: Tissue microarray
