# Supplementary material for: Insulin-like growth factor 1 receptor affects the survival of primary prostate cancer patients depending on TMPRSS2-ERG status
Source: BMC Cancer. 2017 May 25;17:367. doi: 10.1186/s12885-017-3356-8 (PMC5445474; doi:10.1186/s12885-017-3356-8)
Supplement: Supplementary file 2 — Association between IGF system components and clinico-pathological parameters according to Fisher’s or chi-square tests (when more than 2 categories were present) in 270 cases. (DOC 39 kb) [file 12885_2017_3356_MOESM2_ESM.doc]

**Additional file 2**

**Association between IGF system components and clinico-pathological parameters according to Fisher’s or Chi-square tests (when more than 2 categories were present) in 270 cases.**

| **Parameter** | **p-value** | | | |
| --- | --- | --- | --- | --- |
|  | *IGFBP-3* | *IGF-1* | *IGF-1R* | *INSR* |
| Age^#^ | 0.931 | 0.532 | 0.283 | 0.572 |
| Gleason-sp^#^ | 0.908 | < 0.0001 | 0.063 | 0.814 |
| PSA^#^ | 0.117 | 0.01 | 0.115 | 0.152 |
| cT | 0.793 | 0.001 | 0.429 | 0.591 |
| pT | 0.398 | 0.005 | 0.573 | > 0.999 |
| pN* | > 0.999 | < 0.0001 | 0.158 | 0.183 |
| Margins | 0.007 | 0.205 | > 0.999 | 0.320 |
| TMPRSS2/ERG | 0.138 | 0.883 | 0.008 | 0.652 |

SP, specimen; cT, clinical stage; PSA, prostatic specific antigen; pN, lymphnode pathological stage

*Lymphadenectomy was limited to the obturator fossa in most of the cases at the inclusion period

^#^ Chi-square test
