# Supplementary material for: Insulin-like growth factor 1 receptor affects the survival of primary prostate cancer patients depending on TMPRSS2-ERG status
Source: BMC Cancer. 2017 May 25;17:367. doi: 10.1186/s12885-017-3356-8 (PMC5445474; doi:10.1186/s12885-017-3356-8)
Supplement: Supplementary file 3 — Association between IGF system components and clinico-pathological parameters according to Fisher’s or chi-square tests (when more than 2 categories were present) in T2E-negative cases. (DOC 38 kb) [file 12885_2017_3356_MOESM3_ESM.doc]

**Additional file 3**

**Association between IGF system components and clinico-pathological parameters according to Fisher’s or Chi-square tests (when more than 2 categories were present) in T2E-negative cases.**

| **Parameter** | **p-value** | | | |
| --- | --- | --- | --- | --- |
|  | *IGFBP-3* | *IGF-1* | *IGF-1R* | *INSR* |
| Age^#^ | 0.008 | 0.796 | 0.791 | 0.540 |
| Gleason-sp^#^ | 0.809 | 0.099 | 0.441 | 0.3 |
| PSA^#^ | 0.803 | 0.022 | 0.422 | 0.989 |
| cT | 0.725 | < 0.0001 | > 0.999 | 0.704 |
| pT | > 0.999 | 0.091 | 0.05 | > 0.999 |
| pN* | > 0.999 | 0.037 | 0.631 | > 0.999 |
| Margins | 0.809 | 0.809 | > 0.999 | 0.013 |

P, specimen; cT, clinical stage; PSA, prostatic specific antigen; pN, lymphnode pathological stage

*Lymphadenectomy was limited to the obturator fossa in most of the cases at the inclusion period

^#^ Chi-square test
