# Supplementary material for: Insulin-like growth factor 1 receptor affects the survival of primary prostate cancer patients depending on TMPRSS2-ERG status
Source: BMC Cancer. 2017 May 25;17:367. doi: 10.1186/s12885-017-3356-8 (PMC5445474; doi:10.1186/s12885-017-3356-8)
Supplement: Supplementary file 4 — Association between IGF system components and clinico-pathological parameters according to Fisher’s or chi-square tests (when more than 2 categories were present) in T2E-positive cases. (DOC 38 kb) [file 12885_2017_3356_MOESM4_ESM.doc]

**Additional file 4**

**Association between IGF system components and clinico-pathological parameters according to Fisher’s or Chi-square tests (when more than 2 categories were present) in T2E-positive cases.**

| **Parameter** | **p-value** | | | |
| --- | --- | --- | --- | --- |
|  | *IGFBP-3* | *IGF-1* | *IGF-1R* | *INSR* |
| Age^#^ | 0.055 | 0.214 | 0.744 | 0.531 |
| Gleason-sp^#^ | 0.595 | 0.004 | 0.027 | 0.6 |
| PSA^#^ | 0.063 | 0.206 | 0.514 | 0.023 |
| cT | 0.268 | 0.268 | 0.711 | 0.709 |
| pT | 0.299 | 0.037 | 0.863 | 0.603 |
| pN* | 0.631 | 0.004 | 0.626 | 0.176 |
| Margins | 0.023 | 0.116 | 0.731 | > 0.999 |

P, specimen; cT, clinical stage; PSA, prostatic specific antigen; pN, lymphnode pathological stage

*Lymphadenectomy was limited to the obturator fossa in most of the cases at the inclusion period

^#^ Chi-square test
