# Supplementary material for: Insulin-like growth factor 1 receptor affects the survival of primary prostate cancer patients depending on TMPRSS2-ERG status
Source: BMC Cancer. 2017 May 25;17:367. doi: 10.1186/s12885-017-3356-8 (PMC5445474; doi:10.1186/s12885-017-3356-8)
Supplement: Supplementary file 5 — BPFS and clinical PFS log-rank and Cox regression tests in T2E-negative PCa patients analyzed with qRT-PCR. (DOC 80 kb) [file 12885_2017_3356_MOESM5_ESM.doc]

**Additional file 5**

**BPFS and clinical PFS log rank and Cox regression tests in T2E-negative PCa patients analyzed by qRT-PCR.**

| T2E-negative |  | Biochemical Progression | | | |  | | Clinical Progression | | | | |  |
| --- | --- | --- | --- | --- | --- | --- | --- | --- | --- | --- | --- | --- | --- |
| Paramet Parameter | *n* | Events  (% BPFS) | *p*-Univariate | HR (95% CI) | *p*-Multivariate | |  | | Events  (% PFS) | *p*-Univariate | HR (95% CI) | *p*-Multivariate | |
| Age |  |  | 0.108 |  |  | |  | |  | 0.402 |  |  | |
| ≤ 55 | 5 | 1 (80) |  |  |  | |  | | 1 (80) |  |  |  | |
| 56-65 | 22 | 15 (23.9) |  |  |  | |  | | 9 (45.9) |  |  |  | |
| 66-75 | 56 | 27 (29.1) |  |  |  | |  | | 13 (73) |  |  |  | |
| > 75 | 9 | 6 (33.3) |  |  |  | |  | | 3 (37.5) |  |  |  | |
| Gleason-sp: |  |  | 0.001 |  | NS | |  | |  | 0.002 |  | NS | |
| 2-6 | 37 | 15 (43.1) |  |  |  | |  | | 4 (85.7) |  |  |  | |
| 7 | 39 | 21 (39) |  |  |  | |  | | 14 (57) |  |  |  | |
| Greater than 7 | 16 | 13 (0) |  |  |  | |  | | 8 (0) |  |  |  | |
| PSA (ng/ml): |  |  | 0.007 |  | NS | |  | |  | 0.937 |  |  | |
| 10 or less | 53 | 22 (38.3) |  |  |  | |  | | 14 (63) |  |  |  | |
| 10-20 | 26 | 16 (27.6) |  |  |  | |  | | 8 (62.6) |  |  |  | |
| Greater than 20 | 13 | 11 (15.4) |  |  |  | |  | | 4 (68.4) |  |  |  | |
| cT: |  |  | 0.007 |  | 0.019 | |  | |  | 0.192 |  |  | |
| cT2b or less | 81 | 39 (37.4) |  | 1 |  | |  | | 22 (64.8) |  |  |  | |
| cT3a or greater | 11 | 10 (0) |  | 2.54 (1.16-5.55) |  | |  | | 4 (60) |  |  |  | |
| pT: |  |  | 0.005 |  | NS | |  | |  | 0.140 |  |  | |
| pT2 or less | 49 | 21 (38.2) |  |  |  | |  | | 11 (75.7) |  |  |  | |
| pT3 or greater | 43 | 28 (26) |  |  |  | |  | | 15 (52.5) |  |  |  | |
| pN: |  |  | 0.041 |  | NS | |  | |  | 0.280 |  |  | |
| pN0 | 80 | 43 (34) |  |  |  | |  | | 23 (63.3) |  |  |  | |
| pN1 or greater | 6 | 5 (16.7) |  |  |  | |  | | 3 (50) |  |  |  | |
| Margins: |  |  | < 0.0001 |  | < 0.0001 | |  | |  | 0.001 |  | 0.001 | |
| Negative | 40 | 11 (53.9) |  | 1 |  | |  | | 4 (87.6) |  | 1 |  | |
| Positive | 52 | 38 (17.4) |  | 5.34 (2.55-11.1) |  | |  | | 22 (43.7) |  | 7.46 (2.21-25) |  | |
| *IGF-1R* |  |  | 0.016 |  | 0.013 | |  | |  | 0.099 |  |  | |
| Low | 23 | 15 (34.8) |  | 1 |  | |  | | 9 (58.9) |  |  |  | |
| High | 69 | 34 (35.3) |  | 0.41 (0.2-0.82) |  | |  | | 17 (67.2) |  |  |  | |
| *INSR* |  |  | 0.217 |  |  | |  | |  | 0.423 |  |  | |
| Low | 22 | 9 (54.5) |  |  |  | |  | | 5 (75.9) |  |  |  | |
| High | 67 | 38 (25.4) |  |  |  | |  | | 21 (58) |  |  |  | |
| *IGF-1* |  |  | 0.001 |  | 0.045 | |  | |  | 0.012 |  | 0.006 | |
| Low | 22 | 17 (14.9) |  | 1 |  | |  | | 10 (51.1) |  | 1 |  | |
| High | 69 | 31 (38.3) |  | 0.49 (0.24-0.98) |  | |  | | 15 (69.3) |  | 0.32 (0.14-0.71) |  | |
| *IGFBP-3* |  |  | 0.832 |  |  | |  | |  | 0.768 |  |  | |
| Low | 23 | 11 (46.5) |  |  |  | |  | | 5 (75.9) |  |  |  | |
| High | 69 | 38 (31.3) |  |  |  | |  | | 21 (62.1) |  |  |  | |
