# Supplementary material for: Insulin-like growth factor 1 receptor affects the survival of primary prostate cancer patients depending on TMPRSS2-ERG status
Source: BMC Cancer. 2017 May 25;17:367. doi: 10.1186/s12885-017-3356-8 (PMC5445474; doi:10.1186/s12885-017-3356-8)
Supplement: Supplementary file 6 — BPFS and clinical PFS log-rank and Cox regression tests in T2E-positive PCa patients analyzed with qRT-PCR. (DOC 83 kb) [file 12885_2017_3356_MOESM6_ESM.doc]

**Additional file 6**

**BPFS and clinical PFS log rank and Cox regression tests in T2E-positive PCa patients analyzed by qRT-PCR.**

| T2E-positive |  | Biochemical Progression | | | |  | | Clinical Progression | | | | |  |
| --- | --- | --- | --- | --- | --- | --- | --- | --- | --- | --- | --- | --- | --- |
| Paramet Parameter | *n* | Events  (% BPFS) | *p*-Univariate | HR (95% CI) | *p*-Multivariate | |  | | Events  (% PFS) | *p*-Univariate | HR (95% CI) | *p*-Multivariate | |
| Age |  |  | 0.676 |  |  | |  | |  | 0.63 |  |  | |
| ≤ 55 | 10 | 3 (70) |  |  |  | |  | | 2 (78.8) |  |  |  | |
| 56-65 | 59 | 28 (22) |  |  |  | |  | | 20 (51.2) |  |  |  | |
| 66-75 | 82 | 31 (58.9) |  |  |  | |  | | 21 (67.6) |  |  |  | |
| > 75 | 27 | 12 (54.1) |  |  |  | |  | | 5 (68.8) |  |  |  | |
| Gleason-sp: |  |  | < 0.0001 |  | < 0.0001 | |  | |  | 0.01 |  | NS | |
| 2-6 | 72 | 20 (64.3) |  | 1 |  | |  | | 13 (71.2) |  |  |  | |
| 7 | 90 | 42 (23.4) |  | 5.26 (2.39-11.6) | < 0.0001 | |  | | 29 (57.2) |  |  |  | |
| Greater than 7 | 16 | 12 (25) |  | 2.99 (1.48-6.02) | 0.002 | |  | | 6 (52.2) |  |  |  | |
| PSA (ng/ml): |  |  | < 0.0001 |  | 0.004 | |  | |  | 0.021 |  | NS | |
| 10 or less | 102 | 35 (52.8) |  | 1 |  | |  | | 21 (72.3) |  |  |  | |
| 10-20 | 48 | 21 (37.6) |  | 2.77 (1.48-5.12) | 0.001 | |  | | 16 (41.3) |  |  |  | |
| Greater than 20 | 27 | 18 (32.9) |  | 2.47 (1.25-4.87) | 0.009 | |  | | 11 (50.7) |  |  |  | |
| cT: |  |  | 0.075 |  |  | |  | |  | 0.053 |  | NS | |
| cT2b or less | 167 | 68 (45.6) |  |  |  | |  | | 44 (61.6) |  |  |  | |
| cT3a or greater | 10 | 6 (37.5) |  |  |  | |  | | 4 (58.3) |  |  |  | |
| pT: |  |  | < 0.0001 |  | 0.003 | |  | |  | 0.003 |  | 0.004 | |
| pT2 or less | 86 | 22 (73.1) |  | 1 |  | |  | | 14 (79) |  | 1 |  | |
| pT3 or greater | 92 | 52 (21.7) |  | 2.35 (1.34-4.09) |  | |  | | 34 (44.8) |  | 2.47 (1.32-4.6) |  | |
| pN: |  |  | < 0.0001 |  | NS | |  | |  | 0.468 |  |  | |
| pN0 | 156 | 61 (47.8) |  |  |  | |  | | 41 (63.4) |  |  |  | |
| pN1 or greater | 6 | 6 (0) |  |  |  | |  | | 2 (50) |  |  |  | |
| Margins: |  |  | < 0.0001 |  | NS | |  | |  | 0.031 |  | NS | |
| Negative | 97 | 29 (57.9) |  |  |  | |  | | 20 (72.4) |  |  |  | |
| Positive | 81 | 45 (21.5) |  |  |  | |  | | 28 (30.4) |  |  |  | |
| *IGF-1R* |  |  | 0.840 |  |  | |  | |  | 0.198 |  |  | |
| Low | 44 | 17 (60) |  |  |  | |  | | 7 (82.1) |  |  |  | |
| High | 134 | 57 (44.2) |  |  |  | |  | | 41 (57.5) |  |  |  | |
| *INSR* |  |  | 0.905 |  |  | |  | |  | 0.689 |  |  | |
| Low | 44 | 18 (55.7) |  |  |  | |  | | 11 (68.7) |  |  |  | |
| High | 132 | 56 (43.1) |  |  |  | |  | | 37 (59.4) |  |  |  | |
| *IGF-1* |  |  | < 0.0001 |  | 0.005 | |  | |  | 0.035 |  | NS | |
| Low | 44 | 27 (16.5) |  | 1 |  | |  | | 16 (28) |  |  |  | |
| High | 134 | 47 (54.6) |  | 0.47 (0.27-0.79) |  | |  | | 32 (69.8) |  |  |  | |
| *IGFBP-3* |  |  | 0.842 |  |  | |  | |  | 0.898 |  |  | |
| Low | 44 | 18 (55.9) |  |  |  | |  | | 11 (69.4) |  |  |  | |
| High | 134 | 56 (41.6) |  |  |  | |  | | 37 (58.8) |  |  |  | |
