# Supplementary material for: Insulin-like growth factor 1 receptor affects the survival of primary prostate cancer patients depending on TMPRSS2-ERG status
Source: BMC Cancer. 2017 May 25;17:367. doi: 10.1186/s12885-017-3356-8 (PMC5445474; doi:10.1186/s12885-017-3356-8)
Supplement: Supplementary file 7 — BPFS and clinical PFS log-rank and Cox regression tests in primary PCa patients analyzed with IHC. (DOC 72 kb) [file 12885_2017_3356_MOESM7_ESM.doc]

**Additional file 7**

**BPFS and clinical PFS log rank and Cox regression tests in primary PCa patients analyzed by IHC.**

| Total cases |  | Biochemical Progression | | | |  | | Clinical Progression | | | | |  |
| --- | --- | --- | --- | --- | --- | --- | --- | --- | --- | --- | --- | --- | --- |
| Paramet Parameter | *n* | Events  (% BPFS) | *p*-Univariate | HR (95% CI) | *p*-Multivariate | |  | | Events  (% PFS) | *p*-Univariate | HR (95% CI) | *p*-Multivariate | |
| Age |  |  | 0.310 |  |  | |  | |  | 0.658 |  |  | |
| ≤ 55 | 12 | 4 (66.7) |  |  |  | |  | | 2 (82.5) |  |  |  | |
| 56-65 | 72 | 38 (26.1) |  |  |  | |  | | 24 (53.3) |  |  |  | |
| 66-75 | 122 | 50 (45.6) |  |  |  | |  | | 31 (68.6) |  |  |  | |
| > 75 | 33 | 17 (46.8) |  |  |  | |  | | 7 (68.1) |  |  |  | |
| Gleason score: |  |  | < 0.0001 |  | < 0.0001 | |  | |  | < 0.0001 |  | 0.005 | |
| 2-6 | 87 | 26 (57.7) |  | 1 |  | |  | | 12 (78.7) |  | 1 |  | |
| 7 | 123 | 60 (29.6) |  | 3.66 (1.95-6.84) | < 0.0001 | |  | | 41 (57.5) |  | 3.66 (1.59-8.4) | 0.002 | |
| Greater than 7 | 29 | 23 (11.5) |  | 2.03 (1.22-3.37) | 0.006 | |  | | 12 (0) |  | 1.41 (0.74-2.71) | 0.292 | |
| PSA (ng/ml): |  |  | < 0.0001 |  | 0.031 | |  | |  | 0.097 |  |  | |
| 10 or less | 133 | 50 (45.2) |  | 1 |  | |  | | 29 (69) |  |  |  | |
| 10-20 | 69 | 34 (39.4) |  | 2.01 (1.17-3.44) | 0.011 | |  | | 23 (51.5) |  |  |  | |
| Greater than 20 | 36 | 25 (27.7) |  | 1.77 (1.03-3.05) | 0.037 | |  | | 13 (58.5) |  |  |  | |
| cT: |  |  | < 0.0001 |  | 0.006 | |  | |  | 0.042 |  | NS | |
| cT2b or less | 219 | 95 (41.4) |  | 1 |  | |  | | 58 (62.8) |  |  |  | |
| cT3a or greater | 19 | 14 (17.6) |  | 2.36 (1.27-4.38) |  | |  | | 7 (59.7) |  |  |  | |
| pT: |  |  | < 0.0001 |  | NS | |  | |  | 0.008 |  | NS | |
| pT2 or less | 115 | 38 (55.1) |  |  |  | |  | | 22 (77.3) |  |  |  | |
| pT3 or greater | 124 | 71 (23.9) |  |  |  | |  | | 43 (49.1) |  |  |  | |
| pN: |  |  | < 0.0001 |  | 0.008 | |  | |  | 0.278 |  |  | |
| pN0 | 209 | 91 (42.2) |  | 1 |  | |  | | 56 (63.5) |  |  |  | |
| pN1 or greater | 10 | 10 (0) |  | 2.65 (1.29-5.43) |  | |  | | 4 (50) |  |  |  | |
| Margins: |  |  | < 0.0001 |  | 0.001 | |  | |  | < 0.0001 |  | 0.012 | |
| Negative | 116 | 33 (54.8) |  | 1 |  | |  | | 19 (77.9) |  | 1 |  | |
| Positive | 123 | 76 (21.6) |  | 2.14 (1.34-3.4) |  | |  | | 46 (39.3) |  | 2.03 (1.17-3.54) |  | |
| ERG intensity |  |  | 0.962 |  |  | |  | |  | 0.268 |  |  | |
| Low | 105 | 49 (35.3) |  |  |  | |  | | 32 (57.9) |  |  |  | |
| High | 110 | 54 (35.6) |  |  |  | |  | | 29 (62.6) |  |  |  | |
|  |  |  |  |  |  | |  | |  |  |  |  | |
