# Supplementary material for: Insulin-like growth factor 1 receptor affects the survival of primary prostate cancer patients depending on TMPRSS2-ERG status
Source: BMC Cancer. 2017 May 25;17:367. doi: 10.1186/s12885-017-3356-8 (PMC5445474; doi:10.1186/s12885-017-3356-8)
Supplement: Supplementary file 8 — BPFS and clinical PFS log-rank and Cox regression tests in ERG-negative PCa patients analyzed with IHC. (DOC 69 kb) [file 12885_2017_3356_MOESM8_ESM.doc]

**Additional file 8**

**BPFS and clinical PFS log rank and Cox regression tests in ERG-negative PCa patients analyzed by IHC.**

| ERG-negative |  | Biochemical Progression | | | |  | | Clinical Progression | | | | |  |
| --- | --- | --- | --- | --- | --- | --- | --- | --- | --- | --- | --- | --- | --- |
| Paramet Parameter | *n* | Events  (% BPFS) | *p*-Univariate | HR (95% CI) | *p*-Multivariate | |  | | Events  (% PFS) | *p*-Univariate | HR (95% CI) | *p*-Multivariate | |
| Age |  |  | 0.504 |  |  | |  | |  | 0.526 |  |  | |
| ≤ 55 | 4 | 1 (75) |  |  |  | |  | | 0 (-) |  |  |  | |
| 56-65 | 28 | 16 (36.2) |  |  |  | |  | | 9 (51.5) |  |  |  | |
| 66-75 | 55 | 23 (26.9) |  |  |  | |  | | 19 (56) |  |  |  | |
| > 75 | 18 | 9 (47.1) |  |  |  | |  | | 4 (74.2) |  |  |  | |
| Gleason score: |  |  | 0.004 |  | 0.052 | |  | |  | 0.029 |  | NS | |
| 2-6 | 34 | 12 (42.5) |  | 1 |  | |  | | 7 (73.6) |  |  |  | |
| 7 | 56 | 26 (45.1) |  | 2.89 (1.18-7.09) | 0.02 | |  | | 17 (61.2) |  |  |  | |
| Greater than 7 | 15 | 11 (16.7) |  | 2.02 (0.98-4.16) | 0.054 | |  | | 8 (0) |  |  |  | |
| PSA (ng/ml): |  |  | 0.738 |  |  | |  | |  | 0.597 |  |  | |
| 10 or less | 52 | 23 (24.7) |  |  |  | |  | | 18 (46.6) |  |  |  | |
| 10-20 | 33 | 15 (49.6) |  |  |  | |  | | 8 (73.1) |  |  |  | |
| Greater than 20 | 18 | 10 (39.3) |  |  |  | |  | | 5 (68.8) |  |  |  | |
| cT: |  |  | 0.044 |  | NS | |  | |  | 0.111 |  |  | |
| cT2b or less | 47 | 18 (28.7) |  |  |  | |  | | 11 (56.2) |  |  |  | |
| cT3a or greater | 58 | 31 (36.7) |  |  |  | |  | | 21 (55.3) |  |  |  | |
| pT: |  |  | 0.014 |  | NS | |  | |  | 0.292 |  |  | |
| pT2 or less | 43 | 16 (31) |  |  |  | |  | | 12 (68.8) |  |  |  | |
| pT3 or greater | 58 | 33 (34.8) |  |  |  | |  | | 20 (48.1) |  |  |  | |
| pN: |  |  | < 0.0001 |  | 0.003 | |  | |  | 0.430 |  |  | |
| pN0 | 94 | 43 (37.1) |  | 1 |  | |  | | 30 (57.1) |  |  |  | |
| pN1 or greater | 5 | 5 (0) |  | 4.34 (1.64-11.9) |  | |  | | 2 (33.3) |  |  |  | |
| Margins: |  |  | 0.001 |  | 0.006 | |  | |  | 0.008 |  | 0.011 | |
| Negative | 45 | 12 (46.3) |  | 1 |  | |  | | 7 (75.5) |  | 1 |  | |
| Positive | 60 | 37 (30.5) |  | 2.57 (1.30-5.07) |  | |  | | 25 (56.7) |  | 2.97 (1.28-6.89) |  | |
| *IGF-1R* |  |  | < 0.0001 |  | < 0.0001 | |  | |  | 0.020 |  | NS | |
| Low | 26 | 18 (26.3) |  | 1 |  | |  | | 12 (45.4) |  |  |  | |
| High | 79 | 31 (40.7) |  | 0.30 (0.16-0.57) |  | |  | | 20 (62.7) |  |  |  | |
