# Supplementary material for: Insulin-like growth factor 1 receptor affects the survival of primary prostate cancer patients depending on TMPRSS2-ERG status
Source: BMC Cancer. 2017 May 25;17:367. doi: 10.1186/s12885-017-3356-8 (PMC5445474; doi:10.1186/s12885-017-3356-8)
Supplement: Supplementary file 9 — BPFS and clinical PFS log-rank and Cox regression tests in ERG-positive PCa patients analyzed with IHC. (DOC 71 kb) [file 12885_2017_3356_MOESM9_ESM.doc]

**Additional file 9**

**BPFS and clinical PFS log rank and Cox regression tests in ERG-positive PCa patients analyzed by IHC.**

| ERG-positive |  | Biochemical Progression | | | |  | | Clinical Progression | | | | |  |
| --- | --- | --- | --- | --- | --- | --- | --- | --- | --- | --- | --- | --- | --- |
| Paramet Parameter | *n* | Events  (% BPFS) | *p*-Univariate | HR (95% CI) | *p*-Multivariate | |  | | Events  (% PFS) | *p*-Univariate | HR (95% CI) | *p*-Multivariate | |
| Age |  |  | 0.884 |  |  | |  | |  | 0.450 |  |  | |
| ≤ 55 | 7 | 3 (57.1) |  |  |  | |  | | 2 (68.6) |  |  |  | |
| 56-65 | 39 | 21 (0) |  |  |  | |  | | 14 (45.3) |  |  |  | |
| 66-75 | 51 | 23 (50.9) |  |  |  | |  | | 10 (77) |  |  |  | |
| > 75 | 13 | 7 (46.2) |  |  |  | |  | | 3 (57.7) |  |  |  | |
| Gleason score: |  |  | < 0.0001 |  | < 0.0001 | |  | |  | 0.002 |  | 0.016 | |
| 2-6 | 41 | 12 (59.2) |  | 1 |  | |  | | 4 (80.7) |  | 1 |  | |
| 7 | 58 | 33 (18) |  | 8.13 (3-22.2) | < 0.0001 | |  | | 23 (49) |  | 2.22 (0.35-13.8) | 0.392 | |
| Greater than 7 | 11 | 9 (18.2) |  | 2.92 (1.25-6.80) | 0.013 | |  | | 2 (74.1) |  | 0.46 (0.10-2.16) | 0.333 | |
| PSA (ng/ml): |  |  | < 0.0001 |  | < 0.0001 | |  | |  | 0.016 |  | 0.019 | |
| 10 or less | 63 | 23 (46.6) |  | 1 |  | |  | | 10 (80.3) |  | 1 |  | |
| 10-20 | 31 | 17 (30.5) |  | 4.56 (2.15-9.7) | < 0.0001 | |  | | 13 (36.3) |  | 2.12 (0.72-6.25) | 0.171 | |
| Greater than 20 | 16 | 14 (0) |  | 2.09 (0.95-4.6) | 0.064 | |  | | 6 (53.3) |  | 0.64 (0.22-1.81) | 0.402 | |
| cT: |  |  | < 0.0001 |  | 0.015 | |  | |  | 0.003 |  | 0.025 | |
| cT2b or less | 34 | 7 (73.3) |  | 1 |  | |  | | 2 (92.5) |  | 1 |  | |
| cT3a or greater | 75 | 47 (24.3) |  | 3.03 (1.24-7.4) |  | |  | | 27 (51.7) |  | 5.26 (1.22-22.7) |  | |
| pT: |  |  | 0.004 |  | NS | |  | |  | 0.051 |  | NS | |
| pT2 or less | 52 | 18 (63.3) |  |  |  | |  | | 9 (80) |  |  |  | |
| pT3 or greater | 58 | 36 (12.7) |  |  |  | |  | | 20 (47.8) |  |  |  | |
| pN: |  |  | 0.001 |  | NS | |  | |  | 0.563 |  |  | |
| pN0 | 91 | 42 (39.1) |  |  |  | |  | | 22 (65.6) |  |  |  | |
| pN1 or greater | 5 | 5 (0) |  |  |  | |  | | 2 (60) |  |  |  | |
| Margins: |  |  | < 0.0001 |  | NS | |  | |  | 0.126 |  |  | |
| Negative | 54 | 18 (51.3) |  |  |  | |  | | 11 (74.8) |  |  |  | |
| Positive | 56 | 36 (15.7) |  |  |  | |  | | 18 (31.5) |  |  |  | |
| *IGF-1R* |  |  | 0.455 |  |  | |  | |  | 0.193 |  |  | |
| Low | 27 | 14 (40.3) |  |  |  | |  | | 4 (82.3) |  |  |  | |
| High | 83 | 40 (37.4) |  |  |  | |  | | 25 (58.4) |  |  |  | |
