# Supplementary material for: Insulin-like growth factor 1 receptor affects the survival of primary prostate cancer patients depending on TMPRSS2-ERG status
Source: BMC Cancer. 2017 May 25;17:367. doi: 10.1186/s12885-017-3356-8 (PMC5445474; doi:10.1186/s12885-017-3356-8)
Supplement: Supplementary file 10 — Association between IGF-1R and clinico-pathological parameters according to Fisher’s or chi-square tests (when more than 2 categories were present) in ERG-negative cases. (DOC 32 kb) [file 12885_2017_3356_MOESM10_ESM.doc]

**Additional file 10**

**Association between IGF-1R and clinico-pathological parameters according to Fisher’s or Chi-square tests (when more than 2 categories were present) in ERG-negative cases.**

| **Parameter** | **p-value** |
| --- | --- |
| Age^#^ | 0.937 |
| Gleason-sp^#^ | 0.215 |
| PSA^#^ | 0.611 |
| cT | 0.502 |
| pT | 0.042 |
| pN* | 0.329 |
| Margins | 0.176 |

P, specimen; cT, clinical stage; PSA, prostatic specific antigen; pN, lymphnode pathological stage

*Lymphadenectomy was limited to the obturator fossa in most of the cases at the inclusion period

^#^ Chi-square test
