# Supplementary material for: Insulin-like growth factor 1 receptor affects the survival of primary prostate cancer patients depending on TMPRSS2-ERG status
Source: BMC Cancer. 2017 May 25;17:367. doi: 10.1186/s12885-017-3356-8 (PMC5445474; doi:10.1186/s12885-017-3356-8)
Supplement: Supplementary file 11 — Association between IGF-1R and clinico-pathological parameters according to Fisher’s or chi-square tests (when more than 2 categories were present) in ERG-positive cases. (DOC 32 kb) [file 12885_2017_3356_MOESM11_ESM.doc]

**Additional file 11**

**Association between IGF-1R and clinico-pathological parameters according to Fisher’s or Chi-square tests (when more than 2 categories were present) in ERG-positive cases.**

| **Parameter** | **p-value** |
| --- | --- |
| Age^#^ | 0.258 |
| Gleason-sp^#^ | 0.206 |
| PSA^#^ | 0.428 |
| cT | 0.238 |
| pT | 0.660 |
| pN* | > 0.999 |
| Margins | 0.271 |

P, specimen; cT, clinical stage; PSA, prostatic specific antigen; pN, lymphnode pathological stage

*Lymphadenectomy was limited to the obturator fossa in most of the cases at the inclusion period

^#^ Chi-square test
